# Cutaneous Manifestations of Human and Murine Leishmaniasis

**DOI:** 10.3390/ijms18061296

**Published:** 2017-06-18

**Authors:** Breanna M. Scorza, Edgar M. Carvalho, Mary E. Wilson

**Affiliations:** 1 Interdisciplinary Graduate Program in Immunology, University of Iowa, Iowa City, IA 52242, USA; breanna-scorza@uiowa.edu; 2 Instituto de Pesquisa Gonçalo Moniz, Fundação Oswaldo Cruz (FIOCRUZ), Salvador, Bahia 40296-710, Brazil; edgar@ufba.br; 3 Federal University of Bahia, Hospital Universitário Prof. Edgard Santos, Salvador, Bahia 40110-060, Brazil; 4 Department of Internal Medicine, University of Iowa, Iowa City, IA 52242, USA; 5 Department of Microbiology, University of Iowa, Iowa City, IA 52242, USA; 6 Veterans’ Affairs Medical Center, Iowa City, IA 52242, USA

**Keywords:** *Leishmania*, cutaneous leishmaniasis, mucosal leishmaniasis, diffuse leishmaniasis, disseminated leishmaniasis, DTH-delayed type hypersensitivity skin test, LST-*Leishmania* skin test, post-kala-azar dermal leishmaniasis, kala-azar, skin immunology

## Abstract

The leishmaniases are diseases caused by pathogenic protozoan parasites of the genus *Leishmania.* Infections are initiated when a sand fly vector inoculates *Leishmania* parasites into the skin of a mammalian host. *Leishmania* causes a spectrum of inflammatory cutaneous disease manifestations. The type of cutaneous pathology is determined in part by the infecting *Leishmania* species, but also by a combination of inflammatory and anti-inflammatory host immune response factors resulting in different clinical outcomes. This review discusses the distinct cutaneous syndromes described in humans, and current knowledge of the inflammatory responses associated with divergent cutaneous pathologic responses to different *Leishmania* species. The contribution of key hematopoietic cells in experimental cutaneous leishmaniasis in mouse models are also reviewed and compared with those observed during human infection. We hypothesize that local skin events influence the ensuing adaptive immune response to *Leishmania* spp. infections, and that the balance between inflammatory and regulatory factors induced by infection are critical for determining cutaneous pathology and outcome of infection.

## 1. Introduction

Leishmaniasis is the group of diseases initiated through the bite of a female phlebotomine sand fly vector. Leishmaniasis is endemic in over 98 countries, and constitutes a widespread disease burden affecting many regions of the world [1]. With the exception of reported cases passed vertically from mother to child or, in dogs in the USA, from female dog to pups [2], *Leishmania* spp. protozoa are inoculated into mammalian skin during a sand fly blood meal at the onset of infection. Although most *Leishmania* spp. are introduced through the skin, the clinical syndrome caused by these protozoa can be widespread, influenced most heavily by the identity of the infecting species. Most fatalities are due to visceral leishmaniasis, in which *Leishmania* spp. parasites disseminate from the site of skin infection to visceral organs where they can cause pathology [3].

The most common form of leishmaniasis is localized cutaneous leishmaniasis (LCL), one type within a larger category of tegumentary leishmaniasis. During uncomplicated LCL, parasites remain localized in skin tissue and lead to chronic slowly healing skin ulcers. The lesions of LCL are painless and can self-heal without treatment. However, disease resolution can take several months and leave disfiguring scars [3]. In 2013, the World Health Organization (WHO) reported an estimated one million cases of LCL within the previous five years [1]. These estimates are likely vast underestimates [4]. Furthermore, the incidence is subject to fluctuation due to localized factors including population displacement events [5]. Soldiers and tourists traveling to endemic areas are at risk for acquiring leishmaniasis and major outbreaks have occurred in the US military [6].

A range of cutaneous manifestations occurs due to *Leishmania* infection. The species of parasite is the most important determinant of clinical outcome. Species of *Leishmania* that typically cause the different clinical forms of tegumentary leishmaniasis in humans are listed in Table 1.

There is considerable variety in disease manifestations beyond merely the species identity. Even within a species it has been difficult to discern the determinants that lead to different disease manifestations. Identification of parasite genetic determinants affecting phenotype has been hampered by the high degree of homology between *Leishmania* species. Genome sequences from multiple strains within species causing different forms of the disease have shown only a few differences [31]. Furthermore, gene synteny was conserved among 99% of genes and a high degree of amino acid conservation within coding regions (77–92%) was measured during a comparative analysis between three *Leishmania* species that cause divergent disease [31]. Most of the genomic variations between *Leishmania* species have been due to differences in gene copy number and chromosomal duplication events. Beyond that, post-transcriptional modifications have been identified between species [32,33].

In addition to the parasite, host immune response plays a key role in the clinical presentation of leishmaniasis. For instance, subjects with evidence of cell-mediated immunity against *Leishmania* antigen, when infected with *L.* (*L.*) *amazonensis* most often develop LCL. In contrast, patients with impairment of the T cell response often develop an anergic syndrome termed diffuse cutaneous leishmaniasis characterized by multiple nodular lesions full of parasites. Currently, it is not well defined what drives these differences in clinical outcome. It is known that parasite strain, genetically or environmentally determined host immune response, and vector components all contribute [3,34,35,36]. Further investigation is needed to define the contributions of host genetics and microbiota to the cutaneous manifestations of leishmaniasis [37,38,39,40].

Despite their different clinical manifestations, all forms of vector-borne leishmaniasis start with a similar series of events in host skin. These include the initial interactions among *Leishmania* promastigotes, vector components, and host skin cells, all serving to influence the innate immune activation cascade. It has become evident that the sand fly salivary components inoculated with the parasite have potent immunomodulatory properties, some of which are being exploited in vaccine development [34]. Furthermore, the parasite itself forms a gelatinous plug in the sand fly containing filamentous proteophosphoglycan, which has been called promastigote secretory gel (PSG). PSG is also included in the sand fly inoculum, and modulates local immune responses in the skin [41,42].

Following this, *Leishmania* parasites ultimately establish intracellular infection among macrophages, the primary *Leishmania* host cell housing a majority of parasites throughout chronic infection. These local events ultimately lead to presentation of *Leishmania* antigen to T lymphocytes to initiate a T cell-mediated immune response. Research in animal models has pointed towards a role for early tissue signals surrounding these events in polarization of the adaptive immune response, a response that guides the course of ensuing disease. A similar role of early events in human leishmaniasis remains difficult to discern [43,44,45,46,47]. Herein, we review the different types of tegumentary leishmaniasis during infection with different *Leishmania* species, and examine how host cutaneous responses contribute to or protect from disease.

## 2. Nomenclature

The terminologies that describe different clinical forms of leishmaniasis involving the skin can be confusing. Because “cutaneous leishmaniasis” (often abbreviated “CL”) often refers to a specific localized entity described below as Localized Cutaneous Leishmaniasis, “tegumentary leishmaniasis” will be used as an overall term for all forms of leishmaniasis skin involvement. Disseminated forms of tegumentary leishmaniasis called “disseminated cutaneous leishmaniasis” and “diffuse cutaneous leishmaniasis” are very different, but both have been abbreviated as DCL in the literature. Herein we will use the newer term “disseminated leishmaniasis” for disseminated cutaneous leishmaniasis, abbreviated DL. Diffuse cutaneous leishmaniasis is an anergic form sometimes called “anergic diffuse cutaneous leishmaniasis” or ADCL. Herein we will refer to this entity as ADCL, though diffuse cutaneous leishmaniasis is most often abbreviated DCL in the literature. Another disseminated form of leishmaniasis that manifests in mucus membranes is often called mucocutaneous leishmaniasis (abbreviated MCL) because it follows cutaneous lesions. The disease is also called “mucosal leishmaniasis” abbreviated ML, as the simultaneous occurrence of cutaneous and mucosal leishmaniasis is rare and because mucosal disease may develop in the absence of concomitant or previous LCL [48]. ML is the appropriate nomenclature for mucosal disease. Just as CL and LCL refer to the same clinical entity, MCL and ML are also interchangeable terms. Additionally, throughout the text (*L.*) denotes *Leishmania* species within the *Leishmania* subgenus and (*V.*) denotes *Leishmania* species within the *Viannia* subgenus.

## 3. Localized Cutaneous Leishmaniasis

LCL is the most prevalent clinical manifestation of leishmaniasis. It is generally non-life threatening but can be socially stigmatizing. LCL can result from infection with any one of a number of *Leishmania* species (Table 1). LCL symptoms develop an estimated 2–8 weeks after the bite of an infected sand fly, depending on the infecting species [49,50,51]. After the asymptomatic incubation period, a single or small number of nodular or papular lesions develop at the site of parasite deposition by the sand fly. Lymphadenopathy can develop as the earliest indication of disease caused by some species (notably *L.* (*V.*) *braziliensis*), and can precede lesion formation [52]. The lesions may progress into well-delimited ulcers with raised edges [3]. LCL can present with open lesions (wet lesions), which are subject to superficial secondary infection by bacterial or fungal species, with *Staphylococcal* species being most common [53]. For reasons that are still under debate, lesion ulceration has been associated lesion healing [54]. Indeed, patients infected with *L.* (*V.*) *braziliensis* who were treated before ulceration had a higher treatment fail rate compared to those treated after ulceration (75% vs. 25.8%) [54]. Lesions are capable of self-healing without treatment in many cases. However, resolution can take several months to years, and results in formation of a depressed, hypopigmented scar. Lesion resolution does not correspond to a sterile cure, as parasites or parasite DNA can be found in the scars of healed patients, years after successful treatment with clinical cure [55,56]. This propensity of parasites to persist in healthy tissue could have implications for many of the other forms of cutaneous leishmaniasis discussed later in this review.

Control of *Leishmania* replication is associated with development of *Leishmania*-specific T lymphocytes producing tumor necrosis factor (TNF) and interferon gamma (IFNγ). IFNγ promotes parasite control by activating infected macrophages, the primary host cell, to produce microbicidal effectors that can kill intracellular parasites [57]. Factors antagonizing the effects of these cytokines, such as interleukin (IL)-10 and transforming growth factor beta (TGFβ), are associated with lack of parasite control [57,58,59]. Active LCL in humans has been associated with a vigorous immune response, which can range from a predominantly type 1 immune response to a mixed type 1 and type 2 response [12,60,61]. Transcripts encoding innate mediators in lesional skin include IL-1β, IL-8, monocyte chemoattractant protein 1 (MCP1), and inducible nitric oxide synthase (iNOS) [8]. The T cell chemoattractants, chemokine (C-X-C motif) ligand 9 (CXCL9) and CXCL10, were the two most highly up regulated transcripts seen in LCL lesions compared to healthy skin in one study [62]. A T helper (T_H_)17-type signature was not observed in LCL lesions in this study, an observation relevant to the localized form of disease [63,64]. Activity of arginase, an enzyme expressed in both non-classically activated macrophages and in neutrophils [65], is also significantly up regulated in LCL lesions compared with healthy human skin [65,66]. After encountering parasites, monocyte-derived macrophages (MDMs) derived from LCL patients increase expression of CXCL8, CCL2, and CXCL9 in vitro, chemokines that recruit neutrophils, monocytes, and activated T cells, respectively; all of which may contribute to lesion formation [67]. Despite increased chemokine production and higher production of superoxide anion than cells from healthy subjects, MDMs from LCL subjects did not differ in their ability to control intracellular *L.* (*V.*) *braziliensis* infection, compared with healthy subject monocytes [67].

Subclinical infection is common in LCL and other forms of leishmaniasis, and can be detected by a lack of lesions or scars suggestive of healed CL, and lack of a history of chronic ulcer but a positive delayed type hypersensitivity (DTH) skin response to leishmanial antigen, or production of IFNγ by blood cells stimulated with *Leishmania* antigen, indicative of a functional cellular immune response [68,69,70]. However, the type 1 (T_H_1-type) response formed by asymptomatic hosts is not as robust as those who develop active disease, with smaller DTH induration, lower levels of antigen-induced IFNγ and TNF production by peripheral blood mononuclear cells (PBMCs), and higher IL-10 production [71]. What drives individuals to present with LCL or remain subclinically infected is not clear. Variation in basal T cell reactivity to *Leishmania* antigens may contribute. Naïve PBMCs from cohorts of healthy volunteers exposed to *L.* (*V.*) *braziliensis* or *L.* (*L.*) *amazonensis* antigen generate responses that can be stratified into high-IFNγ producing and low-IFNγ producing groups [72,73]. Monocyte-derived macrophages isolated from subclinically infected subjects controlled intracellular parasite replication better than macrophages from patients with active LCL, suggesting intrinsic differences in these innate immune cells even in the absence of T cells [67].

**Figure 1:**
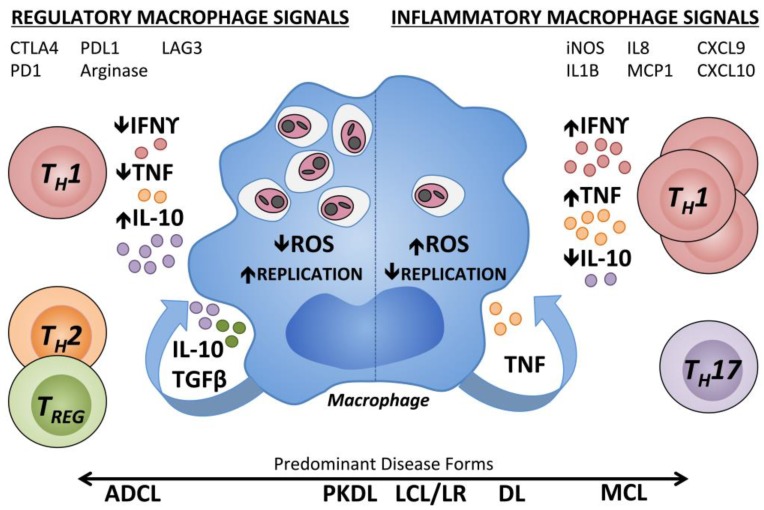
Immune regulation of human macrophages contributing to different pathologic states during *Leishmania* infection. Human macrophages parasitized by *Leishmania* spp. are subject to regulation by cytokines present within the skin at the infection site. Inflammatory Type 1 cytokines, IFNγ and TNF, can synergistically induce ROS production by macrophages, thereby inhibiting *Leishmania* replication within the phagolysosome. T_H_17 cells are increased in inflammatory MCL lesions. Chemoattractant transcripts (IL8, MCP1, CXCL9, and CXCL10) and transcripts associated with classical (type 1) macrophage activation (iNOS, IL1β) have been measured in inflammatory CL lesions. In a regulatory environment (left), there are low levels of type 1 cytokines with lack of microbicidal effectors. T cell or macrophage-derived cytokines including type 2 cytokines, IL-10 and TGFβ antagonize the effects of IFNγ and TNF, and enhanced polyamines can result in parasite proliferation. T_REG_ cells can suppress the effects of IFNγ. Inhibitory receptors on CD4^+^ or CD8^+^ T cells (CTLA4, PD1, and LAG3) and their counter-ligands (CD80, CD86, and PDL1) are associated with T cell exhaustion. Arginase activity is associated with M2-type non-classical macrophage activation. IFNγ: Interferon γ; TNF: Tumor necrosis factor; IL10: Interleukin 10; TGFβ: Transforming growth factor β; ROS: Reactive oxygen species; ADCL: Anergic diffuse cutaneous leishmaniasis; PKDL: Post Kala-Azar dermal leishmaniasis; LCL: Localized cutaneous leishmaniasis; LR: *Leishmania* recidivans; DL: Disseminated leishmaniasis; MCL: Mucocutaneous leishmaniasis; DTH: Delayed type 1 hypersensitivity; T_REG_: T regulatory cell; T_H_: T helper cell; MCP: Monocyte chemoattractant protein; CXCL: Chemokine (C-X-C motif) ligand; CTLA4: Cytotoxic T-lymphocyte associated protein 4; PD1: Programmed cell death protein 1; LAG3: Lymphocyte activation gene 3; PDL1: Programmed death ligand 1.

It has become clear that the outcome of tegumentary infections with the *Leishmania* spp. result from a balance between pro- and anti-inflammatory factors (Figure 1). In infected patients, tissue pathology is associated with a vigorous type 1 immune response to parasite antigens. Lesion size directly correlates with the magnitude *Leishmania* antigen-stimulated TNF production by PBMCs, and with the frequency of circulating TNF and IFNγ producing CD4^+^ lymphocytes [74,75]. During LCL, T cell-derived TNF and IFNγ production are observed by direct staining of early lesions and IL-10 and TGFβ transcripts increase in later stages, perhaps to curb the effects of these inflammatory cytokines once disease has been resolved [76,77]. Similarly, T regulatory cell (T_REG_) transcript forkhead box P3 (FOXP3) is increased intralesionally during chronic LCL due to *L.* (*V.*) *guyanensis* [78]. T_REGS_ from these patients suppressed IFNγ production by autologous CD4^+^ T cells in vitro. The expression of these pro- and anti-inflammatory cytokines by LCL patient PBMCs is sensitive to the local cytokine environment [61]. In circulating monocytes of LCL patients, there is a positive correlation between TNF and IL-10, suggesting a balance between these co-regulatory mechanisms [79]. It may be that clinically overt disease results when the balance between these opposing factors becomes skewed, including LCL and the more extreme atypical manifestations discussed in detail in the following sections.

Recently, RNA-seq of LCL patient biopsies revealed lesional skin containing detectable *L.* (*V.*) *braziliensis* transcripts had a unique transcriptional signature compared with lesional skin that did not contain *L.* (*V.*) *braziliensis* transcripts [80]. Although inflammatory gene transcripts (IFNG and TNF) were increased in these lesions indicative of an active adaptive response, there was a concomitant increase in transcripts encoding inhibitory molecules (IL10, CTLA4, PD1, PDL1, and LAG3). Interestingly, parasite transcript-positive skin was also associated with a significant increase in B cell transcripts (CD79A, CD19, and CD20), suggesting B cell infiltration during active infection [80]. This is supported by another study showing an increase in B cells in *L.* (*V.*) *braziliensis* infected patient lymph nodes during the progression from early pre-lesional to lesional phases of diseases [81]. B cells are able to present *Leishmania* antigen to CD4^+^ T cells and elicit both pro-inflammatory and regulatory cytokine production [82]. Further, *L.* (*V.*) *braziliensis* specific IgG levels are high during the more severe Mucosal Leishmaniasis and decrease with time post treatment [83]. Thus, B cells may contribute to disease onset and persistence during CL.

The role of CD8^+^ T cells in the pathology of LCL has been well documented. While there is no association between CD8^+^ T cell activation and the inflammatory response in the pre-ulcerative phase of the disease, there is an association between the intensity of the inflammation and the frequency of CD8^+^ T cells expressing granzyme [84]. Although cytotoxic activity is higher in LCL than in healthy subjects or in subclinical individuals, CD8^+^ T cells kill *L.* (*V.*) *braziliensis* infected cells but fail to kill intracellular parasites [85,86].

## 4. Mucosal Leishmaniasis

Mucosal leishmaniasis [13] can occur as a complication of LCL caused by *Leishmania* species belonging to the *Viannia* subgenus. In regions where *L.* (*V.*) *braziliensis* is endemic, approximately 1–10% of LCL patients progress to ML. Known risk factors include sex (male > female), increased age, malnutrition, size and number of LCL lesions, lesions above the belt, and inadequate therapy [87,88,89]. ML can occur simultaneously or months–years after resolution of primary lesions, suggesting persistence of latent organisms in the interval [87,88,90]. In this form of tegumentary leishmaniasis, metastatic lesions appear in the mucosal surfaces of the upper respiratory and digestive tracts. Affected areas can include the nasal mucosa, soft palate, pharynx, larynx, lips or cheeks, and rarely, the trachea or genitalia [3]. ML is most prevalent in South America, typically caused by New World species of the *Viannia* subgenus, with *L.* (*V.*) *braziliensis* causing the highest number of cases [17,91,92]. ML is more difficult to treat than LCL, often requiring secondary therapy [93,94,95].

**Figure 2:**
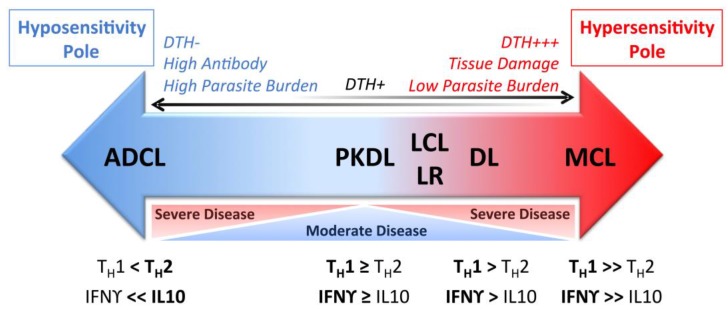
Schematic representation of the spectrum of human cutaneous leishmaniasis manifestations. The localized disease LCL results in the presence of an IFNγ-dominated response, detected by DTH response and T cells. In MCL, T cell responsiveness to *Leishmania* antigen further increases towards the hypersensitivity pole, resulting in tissue damage but low parasite burden. The opposite polar responses with low or absent T cell responsiveness, high parasite burden and antibody titers culminate in ADCL. ADCL: Anergic diffuse cutaneous leishmaniasis; PKDL: Post Kala-Azar dermal leishmaniasis; LCL: Localized cutaneous leishmaniasis; LR: *Leishmania* recidivans; DL: Disseminated leishmaniasis; MCL: Mucocutaneous leishmaniasis; DTH: Delayed type 1 hypersensitivity; T_H_: T helper cell; T_H_1: T_H_1-type immune response; T_H_2: T_H_2-type immune response; IFNγ: Interferon gamma; IL-10: Interleukin 10.

When it is allowed to progress, ML can be highly disfiguring and accompanied by extensive tissue destruction. Lesions contain active parasite-responsive T cells and other inflammatory cells, but the actual burden of detectable parasites is low [96]. Compared with LCL patients, *Leishmania* antigen-stimulated TNF and IFNγ responses of PBMCs are elevated, while IL-10 expression is reduced [97]. ML lesions tend to have higher IL-17 expression than LCL lesions, an inflammation-inducing cytokine, and T_H_17 cells, which may be involved in ML pathogenesis [63,64]. ML patients also have a higher percentage of activated CD4^+^ T cells expressing TNF and IFNγ in circulation compared with LCL patients and, contrary to LCL patients, ML patients do not show a positive correlation between the frequency of TNF^+^ monocytes and IL-10^+^ monocytes [79]. As a result, the ratio of inflammatory to regulatory cytokines is skewed towards the hypersensitivity pole during ML (Figure 2). Targeting this excess TNF for inhibition by pentoxifylline has shown promise in treating patients with refractory ML when combined with standard antimonial therapy [94]. PBMCs from ML patients are also less responsive to IL-10 regulation compared with LCL PBMCs [97] and ML patients have reduced intralesional IL-10 receptor expression [98]. The unregulated inflammatory response in the face of diminished regulatory activity is thought to drive the inflammatory responses leading to tissue destruction in ML. This highlights the importance of immunoregulation in determining the outcome of leishmaniasis.

## 5. Disseminated Leishmaniasis

Although ML was previously the most prevalent metastatic form of cutaneous leishmaniasis due to species of the *Leishmania Viannia* subgenus, disseminated leishmaniasis (DL, also called disseminated cutaneous leishmaniasis or DCL) has emerged as highly prevalent in some regions [12]. DL is characterized by high numbers of pleomorphic lesions, potentially numbering in the hundreds, in two or more noncontiguous anatomical regions [14,99]. Lesions are a mixture of acneiform, papular, nodular, and ulcerated types [25,27]. Parasite metastasis from the original infection site in DL is rapid, commonly occurring within weeks or even days of initial lesion formation [24,27]. The rapidity and breadth of dissemination, and the absence of lymph node enlargement, suggests bloodstream involvement in parasite spread during DL. Nasal mucosal lesions similar to the lesions of ML occur in up to 44% of DL cases [100]. Patients can respond to treatment [26], but may require multiple or slightly longer treatment regimens than LCL treatment recommendations [25,27,100]. Peripheral blood lymphocytes from DL patients produce lower levels of Th1 cytokines than CL patients [31,75,76]. However, at the lesion site, the immune response of DL patients is just as vigorous as lesion cells during LCL, as though a synchronous migration of antigen reactive cells to the multiple cutaneous lesions left a paucity of antigen-responsive cells in the blood [100]. One important characteristic of DL is the high inflammatory response that is mediated by the parasite strain itself. *L.* (*V.*) *braziliensis* isolates from DL patients induce higher production of TNF and IFNγ than *L.* (*V.*) *braziliensis* isolates from LCL patients [79]. Although the biochemical basis by which DL strains elicit a more vigorous cytokine response than LCL strains remains unknown, amplification of anonymous genetic markers from the genomes of different isolates shows evidence that *L.* (*V.*) *braziliensis* isolates causing different disease forms differ at a genomic level [101].

Some studies indicate DL is becoming more common in South America, and there is a site where it has surpassed ML in incidence in the state of Bahia, northeast Brazil [12]. However, in other regions, the occurrence of DL is still relatively rare [12,27,102]. Although the biochemical basis for differences between *L.* (*V.*) *braziliensis* isolates causing distinct syndromes is unknown, at least in one region genetic variability between strains within a species contributes to the outcome of disease. *L.* (*V.*) *braziliensis* strains isolated from DL vs. LCL patients in the same region of Brazil showed significant enrichment for distinct polymorphisms [102]. Furthermore, the expansion of strains was shown to parallel the geographical clustering of DL cases in areas of Brazil, indicating circulation of a particular parasite strain might account for the local increased incidence of DL [103].

Genetic variability in the human population has been reported as significantly associated with DL [104] or with ML in endemic regions [35,36,105,106,107]. Although some descriptions have confused DL with the anergic diffuse cutaneous leishmaniasis (ADCL), it is evident that DL is distinct clinically, immunologically and pathologically from LCL.

## 6. Anergic Diffuse Cutaneous Leishmaniasis

Patients with LCL, MCL or DL develop a vigorous type 1 immune response to the causative *Leishmania* species, which can be detected by a positive DTH response to *Leishmania* antigen. This contrasts with patients who contract anergic diffuse cutaneous leishmaniasis (ADCL), who have a conspicuous absence of a specific *Leishmania* DTH response (Figure 2).

ADCL (also called diffuse cutaneous leishmaniasis or DCL) is a rare but severe form of LCL, characterized by the development of multiple satellite lesions that can coalesce into plaques covering large areas of skin. Lesions are predominantly nodular or papular in nature, contain an abundance of amastigotes, and do not ulcerate [99]. Lesion histopathology shows numerous parasitized macrophages but few lymphocytes, and hyperkeratosis with epidermal hyperplasia [23]. The lack of ulceration and uncontrolled amastigote growth in ADCL are thought to be a consequence of an anergic cellular immune response. Supporting this hypothesis, ADCL patients have absent DTH and lymphocyte proliferative responses to *Leishmania* antigen [108,109]. Anergy is confined to *Leishmania*-specific cellular immune responses, as responses to unrelated antigens are preserved [25,110,111]. Very much like the responses in visceral leishmaniasis, circulating *Leishmania* specific antibodies are found at high levels in these patients [25,108,112]. Compared to LCL, ADCL lesions have significantly reduced numbers of IFNγ, iNOS, and IL-12 producing cells [113]. These infections are often highly resistant to treatment and exhibit frequent relapse. Cutaneous disease does not self-heal over time as is seen in LCL [108,114,115,116]. Additionally, patients with ADCL have significantly fewer circulating and lesional innate natural killer (NK) cells than LCL patients, and the NK cells present secrete less IFNγ and TNF than LCL NK cells following stimulation with *Leishmania* lipophosphoglycan, a parasite surface glycolipid that can ligate Toll-like receptor 2 [117]. In mouse models, high amounts of persistent antigen exposure have been shown to contribute to T cell anergy [118,119,120]. However, in ADCL it is not known whether immune hyposensitivity leads to the observed high parasite burden or vice versa.

ADCL is rare in areas endemic for transmission of other forms of CL, suggesting there may be a contribution of parasite strain to disease. However, when *L.* (*V.*) *pifanoi* parasites isolated from an ADCL lesion were inoculated into human volunteers, all developed classical LCL [20] suggesting this unusual disease form is caused by more than just the strain of parasite. Conventional chemotherapies affecting parasite growth have limited effects in these patients, perhaps because they do not correct the immune defects [121]. However, some success has been seen using an immunotherapy approach in ADCL patients that previously were unresponsive to treatment [122,123,124]. One goal of studies about mechanisms of human T cell anergy is to direct approaches of immunotherapy that might be used to improve treatment for ADCL patients.

## 7. Leishmaniasis Recidivans

Leishmaniasis recidivans (LR) is a rare, chronic relapsing form of tegumentary leishmaniasis that can occur following primary infection with *L.* (*L.*) *tropica*, *L.* (*L.*) *major* or *L.* (*V.*) *braziliensis*, in decreasing order of frequency [3,125,126]. About 5% of CL patients develop LR in Iran [127]. Disease manifests at variable time periods after resolution of a primary LCL infection, sometimes many years later. One case of late-onset LR occurred 43 years after initial infection, highlighting the ability of the parasite to persist in the human host for long periods of time without symptoms [125]. For unclear reasons, nascent lesions reactivate usually around the border, or adjacent to, the scars of previously healed *Leishmania* lesions [128]. LR lesions are usually painless and contain granulomatous inflammatory infiltrates but do not ulcerate. Amastigotes are rarely observed within lesions by microscopy, and the infecting parasite species can only be confirmed by PCR [126,127,128,129]. Similar to other recurrent or disseminated manifestations, this form of leishmaniasis can be difficult to treat [130].

The proximity of nascent LR lesions to primary lesion sites leads to the hypothesis that a small number of organisms survive silently within the skin at the site of initial infection, and can lead to reactivated inflammatory lesions years after initial cure, responding to an unknown stimulus. The cellular localization of parasites during periods of latency remains unknown. One hypothesis is that local trauma may play a role in reactivation of LR lesions, perhaps via inflammatory changes in the local microenvironment [125,131]. In mice, IL-10 and TGFβ have been implicated as factors promoting the persistence of *L.* (*L.*) *tropica* long after the initial infection subsides, but a role for these cytokines in human LR has not been documented [132].

## 8. Post Kala-Azar Dermal Leishmaniasis

Post Kala-Azar dermal leishmaniasis (PKDL) is a complication of visceral leishmaniasis caused by *L.* (*L.*) *donovani*, primarily occurring in east Africa (Sudan, Ethiopia, Kenya) and the Indian subcontinent (India, Bangladesh, Nepal) [29]. There are numerous clinical presentations of PKDL. In Africa, PKDL presents most commonly as a collection of dermatoses comprising a macular, maculopapular, or nodular rash [28]. In Indian PKDL, erythema and a combination of macular and papular lesions form and may coalesce into plaques [133,134]. PKDL lesions do not typically become ulcerated [135] and persist for several months [30]. PKDL can occur concurrently with visceral leishmaniasis (VL) or following resolution of visceral infection [28]. A subset of PKDL cases (15–20%) occur without any previous symptoms of VL, showing that for unknown reasons asymptomatic infections can convert to PKDL [134]. Fifty to sixty percent of Sudanese VL patients can develop PKDL, whereas the incidence in India is only 5–10% of VL cases [136]. In *L.* (*L.*) *donovani* endemic areas, the instance of PKDL is higher in HIV-positive patients [137]. The onset of PKDL in Africa also has different kinetics compared to Indian PKDL, as it manifests within months of VL treatment, while in India PKDL may take years to appear [29].

Although light microscopic studies show a low abundance of amastigotes within PKDL lesions [135,138], parasites can still be detected in affected skin using specific antibody staining [139,140]. This has lead scientists to hypothesize that subjects with PKDL serve as a reservoir maintaining continued parasite transmission in some regions, particularly in the Indian subcontinent [133]. This is consistent with the observation that VL in India is primarily an anthroponotic disease [141]. Following resolution of PKDL, there is apparent protective immunity against re-developing symptomatic disease, although there are rare cases of relapse [142,143,144]. Although PKDL can spontaneously heal without treatment (average 9.7 months in Sudan) [30], patients may contribute significantly to anthroponotic transmission of the infection. Therefore, it is considered an urgent priority to identify and treat PKDL cases to prevent continuation of VL in the Indian subcontinent [4].

Although most clinicians in India do not use the DTH skin test, studies in Sudan show that DTH skin tests are negative and PBMCs display absent proliferation and IFNγ responses to *Leishmania* antigen during active VL [6,136]. Successful treatment of VL precedes the recovery of T cell reactivity to *Leishmania* antigen, and in subjects who develop PKDL this recovery is coincident with PKDL development [140,145]. PKDL patients show an intermediate level of T cell reactivity between active VL patients and fully recovered patients. It is important to note, however, that only a subset of treated patients develop PKDL, despite restoration of skin test responses in both [28]. This discrepancy may be linked to IL-10 production in PKDL patients. Inadequate treatment of VL increases the probability of developing PKDL [28]. Unlike leishmaniasis recidivans, IL-10 is thought to play a major role in the etiology of PKDL. VL patients who develop PKDL have elevated serum IL-10 and antigen-induced IL-10 production by PBMCs compared to patients who do not develop PKDL [140]. PKDL lesions contain a range of infiltrating cells including numerous CD4^+^ and CD8^+^ cells, and granulomas are present at varying levels of maturity [146]. Concurrent IL-10 and IFNγ gene expression was observed in the lymph nodes, skin lesions [139,147] and skin keratinocytes of subjects with PKDL [140].

One theory is that PKDL develops in part due to the effects of UV light on vitamin D metabolism of local or circulating mononuclear cells. The localization of lesions in affected patients tends to be skewed toward sun-exposed skin surfaces [29,148]. Vitamin D3 synthesis is increased following UV exposure, and this in turn suppresses monocyte activation [149,150]. In addition, PKDL patients have higher amounts of the bioactive form of vitamin D3 in their plasma compared to other VL patients post-treatment [151]. PKDL circulating and intralesional monocyte/macrophages display a phenotype that is not classically activated, and has characteristics of M2-type cells with peroxisome proliferator-activated receptor gamma (PPARy), arginase 1 (ARG1), and CD206 expression [152]. This macrophage activation state was hypothesized to contribute to the persistence of this condition [151,153].

Reports of PKDL due to other visceralizing *Leishmania* species such as *L.* (*L.*) *infantum* are scarce. Some cases of non-ulcerating cutaneous lesions due to *L.* (*L.*) *infantum* infection have been reported, but these constitute a separate disease state from PKDL [154]. PKDL symptomology cannot be recapitulated using rodent models [155].

## 9. Cellular Determinants of the Immune Response in Murine Cutaneous Leishmaniasis

Mouse models have been used extensively to study cutaneous and visceral leishmaniasis [46]. As in human disease, clinical manifestations in mice vary after infection with different *Leishmania* species. Different mouse strains have genetically programmed variations in the severity of infection [156], an observation that has inspired many studies of human genetic susceptibility [157]. In particular, the mouse model of *L.* (*L.*) *major* infection has been widely used to study CL [46]. BALB/c mice are highly susceptible to *L.* (*L.*) *major* infection, developing a T_H_2-type CD4^+^ T cell response, large local ulcerating lesions, and eventually visceral dissemination of parasites. In contrast, C57BL/6, CBA, and C3H mice are relatively resistant to *L.* (*L.*) *major*, developing a T_H_1-type cellular immune response and small lesions that ultimately self-heal without dissemination [158,159,160]. Antigen specific T_H_1-type cells express IFNγ upon contact with antigen presenting cells [161], which in turn activates microbicidal defenses of infected macrophages to kill intracellular parasites [161,162]. Similar to humans, pathology during murine CL is caused by immune-mediated damage. As an illustration, CD4^+^ T cell deficient C57BL/6 mice experience minimal cutaneous pathology despite a high parasite burden in the ear [163]. Furthermore, during both *L.* (*L.*) *major* and *L.* (*L.*) *infantum* murine infection, there is a prolonged period of silent parasite replication during the first weeks of infection, during which pathology is not observed. After approximately four weeks, infiltration of immune cells coincides with the onset of pathologic changes [163,164].

Although murine models of *L.* (*L.*) *major* infection have been useful for elucidating mechanisms controlling type 1 vs. type 2 CD4^+^ T cell polarization, this dichotomy is not observed during murine infection with other *Leishmania* species. Murine infection with *L.* (*L.*) *mexicana* results in a chronic phenotype in most mouse strains [165]. *L.* (*V.*) *braziliensis* forms small, non-ulcerative and self-healing lesions in wild type mice [166]. There is a range of resistance to *L.* (*L.*) *amazonensis* in different mouse strains, with higher levels of antibody being associated with susceptibility [167]. In contrast to *L.* (*L.*) *donovani* and *L.* (*L.*) *infantum*, in which murine susceptibility maps to a single locus encoding solute carrier family 11 member 1 (Slc11a1, previously called Nramp1) on chromosome 1 [168], murine susceptibility to CL is associated with more than one chromosomal locus [168,169,170].

Mouse models of CL show that treated or self-resolved mice can achieve disease resolution, measured by decreased parasite burden and lack of cutaneous lesions, but that parasites persist in the host for long periods of time [171,172], as long as the production of IL-10 is intact [173]. Similar to humans, protective immunity in mouse models follows non-sterilizing cure [171,174]. Both CD4^+^ and CD8^+^ T cells contribute to this protection [175]. The cell types harboring latent parasites include macrophages, dendritic cells, and fibroblasts, although additional cell types may play similar roles [176,177,178]. In mice infected with *L.* (*L.*) *major*, IL-10 blockade at chronic time points leads to sterile cure [132,173]. Intriguingly, *L.* (*L.*) *major*-infected IL-10 deficient mice achieve a sterile cure, but these mice do not develop immunity to reinfection, suggesting that persistent parasites may be integral to achieving long-lasting immunity [179]. Nonetheless, IL-10 impairs cure of acute leishmaniasis, illustrated in IL-10 deficient mice infected with *L.* (*L.*) *amazonensis* or *L.* (*L.*) *mexicana* which are still unable to eliminate infection despite strong IFNγ production [180,181,182], at least in part through the action of IL-4 [181].

Early immune events within a critical 24–48 h post-infection have been implicated in instructing the polarization of the T_H_ response in leishmaniasis [46]. The cytokine context within which dendritic cells (DCs) present antigen to CD4^+^ T cells is one important determinant of development of T_H_0 cells toward either T_H_1 vs. T_H_2 poles [183,184,185]. The signals that induce T cell polarizing cytokine expression are derived from the skin inflammatory milieu in which DCs encounter antigen. During experimental cutaneous leishmaniasis due to *L.* (*L.*) *major*, skin-infiltrating inflammatory monocytes differentiate locally into DCs and subsequently migrate to the draining lymph nodes, where they induce polarized CD4^+^ T cell responses [45]. Thus, the early skin microenvironment has an important role in influencing the adaptive T cell response and the progression or resolution of disease. Mice offer a unique opportunity to dissect early molecular and cellular mechanisms occurring within the site of infection.

### 9.1. Neutrophils

**Figure 3:**
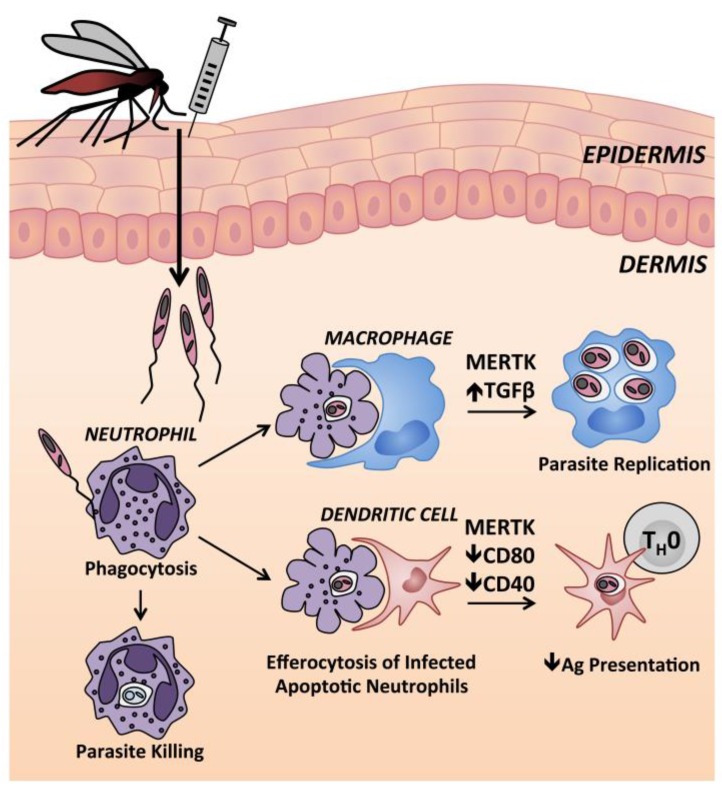
Neutrophils at the cutaneous site of *Leishmania* spp. inoculation in mice. Neutrophils are rapidly recruited to the site of *Leishmania* infection, initiated by either sand fly or needle inoculation, where they phagocytose parasites. Although some *Leishmania* are killed by neutrophils, some survive within neutrophils until they undergo apoptosis. Uptake of apoptotic infected neutrophils by macrophages leads to MERTK signaling and increased TGFβ release, promoting an anti-inflammatory macrophage state and intracellular parasite replication. DCs that take up apoptotic infected neutrophils show a decreased capacity to present antigen to naïve CD4^+^ T cells. MERTK: Mer tyrosine kinase; TGFβ: Transforming growth factor β, T_H_0: naïve CD4^+^ T cell.

Neutrophils are the first immune cells recruited to the site of cutaneous infection after delivery of *L.* (*L.*) *major* to mouse ears [186,187]. There is conflicting evidence on whether neutrophils play a protective or pathogenic role in leishmaniasis. Neutrophils phagocytose parasites in vitro and within infected skin tissue [186,188]. It has been suggested that parasites remain viable within parasitophorous vacuoles after neutrophils undergo apoptosis. Apoptotic neutrophils are engulfed by macrophages in a process called efferocytosis (Figure 3), which induces an anti-inflammatory macrophage state with TGFβ release [189] and signaling through Mer tyrosine kinase receptors [190]. During leishmaniasis, macrophage clearance of apoptotic, infected neutrophils has been likened to a Trojan horse because microbicidal responses are suppressed during macrophage infection. Importantly, apoptotic infected neutrophils are also taken up by dendritic cells, with consequent signaling through the Mer kinase pathway and impaired ability to present antigen to T cells [190]. Dermal DCs that take up infected neutrophils have lowered expression of co-stimulatory receptors CD40 and CD80 [190]. Along these lines, one study showed higher activation of infected dermal DCs and enhanced CD4^+^ T cell priming in neutrophil depleted mice [187].

### 9.2. Langerhans Cells

Langerhans cells (LCs) are a subset of dendritic cells resident in the epidermis whose role during cutaneous leishmaniasis is unclear. It has been demonstrated that epidermal LCs can take up *L.* (*L.*) *major* parasites and migrate from the skin to the draining lymph nodes [191,192]. However, recent imaging studies of intradermally infected mouse skin revealed that *L.* (*L.*) *major* parasites are rapidly taken up by dermal and inflammatory DC subsets, but not by LCs [193,194]. Two studies depleted langerin-expressing cells (which are mostly LCs) using diphtheria toxin receptor transgenic mice, prior to *L.* (*L.*) *major* infection. The first showed no effect on CD4^+^ T cell priming or parasite burden [195], whereas the other showed reduced lesion size and reduced antigen-specific IL-10 production [196]. The data indicate that LCs interact with *Leishmania* spp. and participate in the immune response, although the biological relevance of these cells remains controversial.

### 9.3. Monocytes and Macrophages

**Figure 4:**
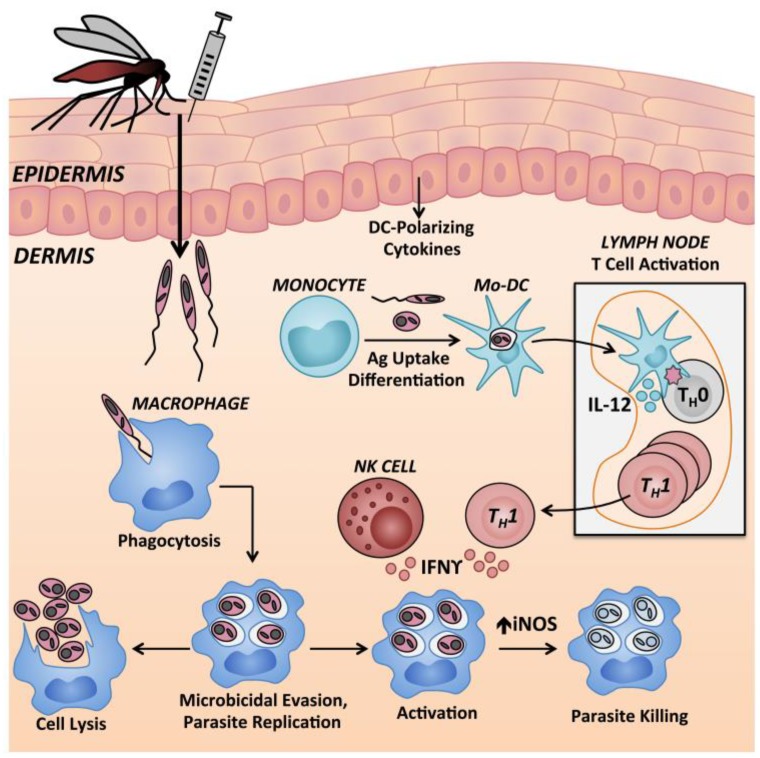
Monocytic cells in control of *Leishmania* replication in murine skin. Epidermal cytokines can modulate this process. Monocyte derived dendritic cells (Mo-DCs) transport antigen to draining lymph nodes for presentation to naïve CD4^+^ T cells and resultant T_H_1 cells traffic back to the site of infection where they secrete IFNγ. Recruited inflammatory monocytes are the primary host cell allowing intracellular survival and replication of *Leishmania* spp. Monocytes recruited to the site of intradermal *Leishmania* inoculation can differentiate locally into dendritic cells or macrophages. Once phagocytosed by macrophages, *Leishmania* use a variety of virulence mechanisms to suppress the host cell microbicidal response and replicate intracellularly. Infected macrophages can either facilitate intracellular survival and/or replication, or if exposed to activating signals such as IFNγ from T_H_1-type cells or NK cells, can upregulate iNOS. The combination of reactive oxygen and reactive nitrogen species can contribute to killing of intracellular parasites. Mo-DC: Monocyte derived dendritic cell; NK cell: Natural killer cell; IFNγ: Interferon γ; IL-12: Interleukin-12; iNOS: Inducible nitric oxide synthase; T_H_1: T helper 1 cell, T_H_0: Naïve CD4^+^ T cell.

Monocytes are recruited to the site of *Leishmania* infection and can internalize parasites (Figure 4). Monocytes differentiated into dendritic cells within the skin environment (termed dermal monocyte-derived DCs) are the major cell type responsible for inducing protective *Leishmania*-specific T_H_1 cells in skin draining lymph nodes [45].

Resident and recruited macrophages harbor the greatest burden of intracellular *Leishmania* spp. parasites throughout chronic leishmaniasis [186,197]. Macrophage infection can be acquired via efferocytosis, as summarized in Figure 3, and mouse and human macrophages internalize free parasites via receptor-mediated phagocytosis [198]. Both phagocytosis and inflammatory cytokines (IL-1, TNF, type 1 interferons) can stimulate macrophages to generate microbicidal reactive oxygen species and reactive nitrogen species, through assembly of the nicotinamide adenine dinucleotide phosphate (NADPH) oxidase or activation of iNOS, respectively [199,200,201,202,203]. Activation requires priming and activation signals, and TNF acts synergistically with IFNγ to induce NO and amastigote elimination by mouse macrophages (Figure 4) [204,205]. In both human and murine macrophages, the respiratory burst is subverted by *Leishmania* virulence factors [206,207]. Phagocytosis of amastigotes induces even less superoxide formation compared with promastigotes [208]. Mechanisms of subversion include inhibition of assembly and maturation of the NADPH oxidase machinery at the phagosomal membrane, antioxidant activity of *Leishmania* enzymes, and active macrophage deactivation through the SH2-domain containing inositol phosphatase 1 (SHIP-1) [203,209].

The relative importance of NADPH oxidase vs. iNOS for macrophage microbicidal responses was explored with knockout mouse models. Macrophages lacking a functional NADPH oxidase were able to kill intracellular *L.* (*V.*) *braziliensis* parasites when stimulated with IFNγ as well as wild-type macrophages, while iNOS-deficient macrophages were unresponsive to IFNγ activation [166]. Additionally, mice normally resistant to *L.* (*L.*) *major* infection lacking iNOS are susceptible, despite eliciting a robust T_H_1 immune response [210]. Macrophage killing mechanisms may be species dependent as both NO and ROS were required by C3H bone marrow-derived macrophages to kill intracellular *L.* (*L.*) *amazonensis* [211]. The extent to which human macrophages express and utilize NO production to combat *Leishmania* infection is controversial [202,212,213].

### 9.4. Natural Killer Cells

NK cells are innate immune effector cells that were shown to be protective against murine *L.* (*L.*) *major* infection [214,215]. NK cells are recruited to the skin infection site and draining lymph nodes within 24 h of *L.* (*L.*) *major* inoculation [216,217]. NK cell activity is higher during the early stages of *L.* (*L.*) *major* infection of resistant mice compared to susceptible mice, and the local parasite burden in the skin is inversely correlated with early NK cytotoxic activity in different mouse strains [215]. Activated NK cells augment IL-12p40 production by *L.* (*L.*) *amazonensis* promastigote-infected DCs [218]. These DC1 cells then preferentially induce T_H_1 polarization of naïve CD4^+^ T cells [215,219]. NK cell-derived IFNγ can also activate local *Leishmania* infected macrophages (Figure 4) but NK-derived IFNγ is insufficient to confer protection in the absence of T_H_1 cells [220,221]. Conversely, TGFβ suppresses NK cell IFNγ production, and blockade of TGFβ signaling in NK cells allows susceptible BALB/c mice to control *L.* (*L.*) *major* infection [222]. Overall, the literature supports a protective, but non-essential, role for NK cells during the early stages of experimental cutaneous leishmaniasis.

### 9.5. Keratinocytes

Other resident cell types critical to immune responses at the site of *Leishmania* spp. infection remain to be fully explored. Keratinocytes reside in the epidermis and participate a communicative immune network in the skin, mediated in part by cytokine exchange with the local cell populations [223]. In a model of *L.* (*L.*) *major*, expression of immunomodulatory mediators involved in T_H_1 induction were significantly highly induced in keratinocytes isolated from the infected skin of resistant mice and several were enriched in the epidermis upon RNA in situ hybridization, suggesting keratinocytes may be a rich source of skin derived cytokines in vivo [44]. Interferon-inducible T-cell alpha chemokine 1 (I-TAC), an early released cytokine that antagonized IL-12 production by DCs, is significantly increased in the epidermis and keratinocytes of susceptible compared to resistant mice infected with *L.* (*L.*) *major* [224]. Experiments performed in our lab suggest *Leishmania* species causing divergent clinical disease elicit distinct inflammatory responses by human keratinocytes in vitro. VL causing *L.* (*L.*) *infantum*-exposed human keratinocytes up-regulate pro-inflammatory cytokine gene expression with concurrent nuclear factor-κB (NF-κB) activation, while CL-causing *L.* (*L.*) *major* may inhibit keratinocyte activation [225]. Together, these studies suggest a role for keratinocyte-derived cytokines in shaping the immune response to *Leishmania*, warranting future investigation in this area.

## 10. Concluding Remarks

The different species of *Leishmania* cause characteristic clinical syndromes. However, there is nonetheless a spectrum of outcomes of infection with each *Leishmania* species in both human leishmaniasis and animal models. The range of clinical outcomes, when understood, seems to depend on key contributions from infecting parasite species and a delicate balance of pro- and anti-inflammatory host immune factors in response to infection. An interesting property of the *Leishmania* species infections is the persistence of parasites long after clinical cure of symptomatic infection, a property that can result in downstream cutaneous manifestations, but which also may be critical for developing immune protection against re-infection. The mechanisms and cell types involved in parasite persistence, and how they lead to disease re-emergence vs. immune protection, remain poorly understood and a subject of great interest for investigators.

Mouse models have revealed early host signals are important controllers of the clinical response to *Leishmania* spp. infection. This has led to the development of improved models that better mimic natural infection conditions. Our group and others have postulated a previously unappreciated role for inflammatory tissue signals derived from resident, non-hematopoietic cells such as keratinocytes, during early stages of *Leishmania* infection within the skin. Additional signals generated from sand fly derived factors also cannot be overlooked. Ongoing investigations aim to determine the cellular targets of these vector elicited effects. Mounting evidence suggests the skin microenvironment has a critical influence on the course of *Leishmania* infection. Therefore, elucidation of early cutaneous inflammatory mechanisms is an important subject of future research in the field of leishmaniasis.

## Figures and Tables

**Table 1:** Major clinical forms of tegumentary leishmaniasis and causative *Leishmania* species.

Clinical Syndrome	Causative Species ^1^	Clinical Manifestation
Localized Cutaneous Leishmaniasis (LCL)	*L.* (*L.*) *major* [3] *L.* (*L.*) *mexicana* [7] *L.* (*L.*) *amazonensis* [7] *L.* (*V.*) *braziliensis* [7] *L.* (*L.*) *tropica* [8] *L.* (*L.*) *aethiopica* [9] *L.* (*V.*) *panamanensis* [7] *L.* (*L.*) *infantum* [10] *L.* (*L.*) *donovani* [11]	Single or limited number of lesions; ulcers formed can be wet or dry with raised crateriform border. Moderate parasite loads in biopsies of the ulcer border; positive DTH ^2^ response [12]
Mucosal Leishmaniasis [13]	*L.* (*V.*) *braziliensis* [14] *L.* (*V.*) *panamanensis* [15] *L.* (*V.*) *guyanensis* [16] *L.* (*L.*) *amazonensis* [14,17]	Highly inflammatory lesions involving mucosal membranes; can be disfiguring. Rare parasite forms present in biopsies; strong DTH response [12]
Anergic Diffuse Cutaneous Leishmaniasis (ADCL)	*L.* (*L.*) *amazonensis* [18] *L.* (*L.*) *mexicana* [19] *L.* (*V.*) *pifanoi* [20] *L.* (*L.*) *aethiopica* [21] *L.* (*L.*) *major* [22]	Multiple, disseminated, non-ulcerative nodular lesions; many parasites in lesions; absent DTH response (anergy) [20,23]
Disseminated Leishmaniasis (DL)	*L.* (*V.*) *braziliensis* [24] *L.* (*V.*) *panamanensis* [25] *L.* (*V.*) *guyanensis* [25,26] *L.* (*L.*) *amazonensis* [24]	Numerous papular/acneiform lesions in ≥2 non-contiguous areas of the body, commonly involving mucosal membranes. Few parasites in lesions; strong DTH response [27]
Post-Kala Azar Dermal Leishmaniasis (PKDL)	*L.* (*L.*) *donovani* [1]	Hypopigmented macular, maculopapular, or nodular rash. Interferon γ (IFNγ) response to *Leishmania* antigens. Parasites are present in lesions [28,29,30]

^1^ (*L.*) denotes *Leishmania* subgenus. (*V.*) denotes *Viannia* subgenus. ^2^ DTH refers to a delayed type hypersensitivity response to *Leishmania* antigen, a test that is also called the *Leishmania* skin test (LST) or Montenegro test.

## Abbreviations

LCL	Localized cutaneous leishmaniasis (also called cutaneous leishmaniasis or CL)
ADCL	Anergic diffuse cutaneous leishmaniasis (also called diffuse cutaneous leishmaniasis or DCL)
DL	Disseminated leishmaniasis (also called disseminated cutaneous leishmaniasis)
LC	Langerhans’ cell
LR	Leishmaniasis recidivans
MDM	Monocyte derived macrophage
MCL	Mucocutaneous leishmaniasis (also called mucosal leishmaniasis or ML)
PBMC	Peripheral blood mononuclear cells
NADPH	Nicotinamide adenine dinucleotide phosphate
